# Folate metabolite profiling of different cell types and embryos suggests variation in folate one-carbon metabolism, including developmental changes in human embryonic brain

**DOI:** 10.1007/s11010-013-1613-y

**Published:** 2013-03-13

**Authors:** Kit-Yi Leung, Sandra C. P. De Castro, Filipe Cabreiro, Peter Gustavsson, Andrew J. Copp, Nicholas D. E. Greene

**Affiliations:** 1 Neural Development Unit and Newlife Birth Defects Research Centre, Institute of Child Health, University College London, 30 Guilford Street, London, WC1N 1EH UK; 2 Department of Genetics, Evolution and Environment, Institute of Healthy Ageing, University College London, London, UK; 3 Department of Molecular Medicine and Surgery, Karolinksa Institutet, Stockholm, Sweden

**Keywords:** Folate, Embryo, Methotrexate, Bacteria, Liquid chromatography tandem mass spectrometry

## Abstract

Folates act as co-factors for transfer of one-carbon units for nucleotide production, methylation and other biosynthetic reactions. Comprehensive profiling of multiple folates can be achieved using liquid chromatography tandem mass spectrometry, enabling determination of their relative abundance that may provide an indication of metabolic differences between cell types. For example, cell lines exposed to methotrexate showed a dose-dependent elevation of dihydrofolate, consistent with inhibition of dihydrofolate reductase. We analysed the folate profile of *E. coli* sub-types as well as cell lines and embryonic tissue from both human and mouse. The folate profile of bacteria differed markedly from those of all the mammalian samples, most notably in the greater abundance of formyl tetrahydrofolate. The overall profiles of mouse and human fibroblasts and mid-gestation mouse embryos were broadly similar, with specific differences. The major folate species in these cell types was 5-methyl tetrahydrofolate, in contrast to lymphoblastoid cell lines in which the predominant form was tetrahydrofolate. Analysis of embryonic human brain revealed a shift in folate profile with increasing developmental stage, with a decline in relative abundance of dihydrofolate and increase in 5-methyl tetrahydrofolate. These cell type-specific and developmental changes in folate profile may indicate differential requirements for the various outputs of folate metabolism.

**Electronic supplementary material:**

The online version of this article (doi:10.1007/s11010-013-1613-y) contains supplementary material, which is available to authorized users.

## Introduction

**Fig. 1:**
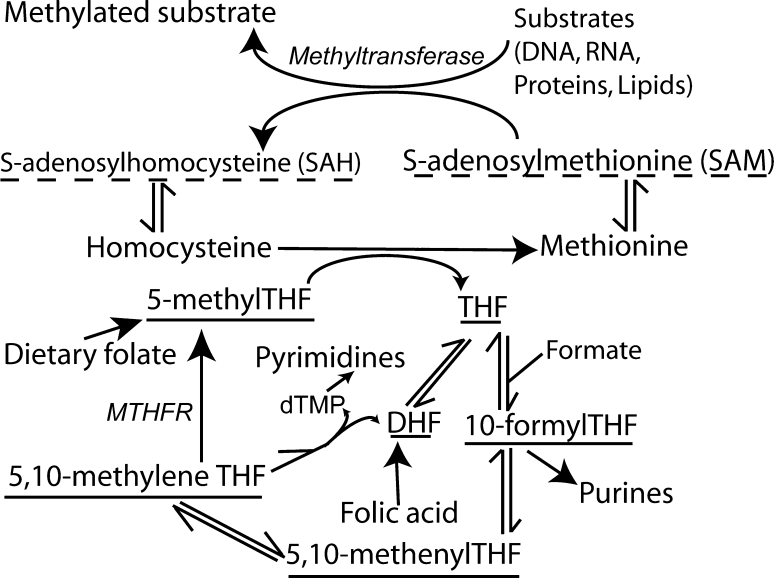
Summary diagram of folate one-carbon metabolism. Folates provide co-factors for the transfer of one-carbon units required for production of pyrimidines, purines and remethylation of homocysteine to methionine. Folates analysed in the current study are underlined, whilst methodology for quantification of SAM and SAH (*dashed underline*) was reported previously [20]

Folate one-carbon metabolism (FOCM) comprises a network of interlinked reactions in which folates act as co-factors for transfer of one-carbon units required in various biosynthetic reactions (Fig. 1) [1–3]. Major functions of FOCM are in production of purines and pyrimidines for biosynthesis of nucleic acids, cysteine production and provision of the methyl group in methylation of DNA, RNA, proteins and lipids as well as synthesis of creatine and phosphatidylcholine. Circulating folate is principally in the form of 5-methyltetrahydrofolate (5-methyl THF). Cellular uptake is mediated by folate receptors (FOLR1 and FOLR2) and the reduced folate carrier (RFC; SLC19a1) and in the gut by the proton-coupled folate transporter (PCFT; SLC46A1)[4]. Embryonic lethality of mice lacking *Folr1* or *Rfc* demonstrates the necessity of folate uptake for postimplantation development [5–7].

Abnormalities in FOCM have been implicated in a variety of pathological conditions including cancer, neural tube defects (NTDs), cardiovascular disease, anaemia and neurological conditions [3, 8–11]. Notably FOCM is implicated in diseases that may occur throughout life, including birth defects that arise during early development. For example, maternal supplementation with folic acid reduces the risk of NTDs whilst sub-optimal folate status and elevated homocysteine are associated with increased predisposition to an affected pregnancy (reviewed in [9]). The observation of defective thymidylate biosynthesis in some human NTD cases and mouse models [12–14] supports the hypothesis that altered FOCM may contribute to development of NTDs. Folate status has also been investigated in relation to risk of several different cancers, colorectal cancer being probably the most intensively studied. Epidemiological studies have shown an association of low folate intake with risk of colorectal adenomas and cancer, and use of folic acid supplements has been reported to reduce risk and mortality. However, folic acid supplements may also promote progression of pre-existing preneoplastic lesions, thereby increasing cancer risk [10, 15]. A similar non-linear relationship, in which both low and high folate intake are associated with risk, has also been reported for postmenopausal breast cancer [16].

The multiple outputs of FOCM imply several different biochemical mechanisms by which impaired function may influence embryonic development and post-natal pathologies. Nucleotide biosynthesis is essential to support DNA replication and cell proliferation, which may be of particular relevance to embryonic development, whilst impaired thymidylate biosynthesis causes an increase in erroneous incorporation of uracil into nuclear DNA [1]. The supply of methyl groups is also required for cellular methylation reactions and may impact DNA methylation, possibly leading to altered transcriptional regulation. In this context, there is increasing evidence of a potential link between FOCM and the foetal epigenome, although whether altered methylation contributes to birth defects has yet to be established [17].

Alterations in FOCM can be investigated by analysis of downstream biomarkers such as homocysteine concentration, DNA methylation and uracil incorporation [1]. Mathematical modelling also allows predictions of the effects of folate status or enzyme inhibition on methylation and nucleotide biosynthesis [18, 19]. It will also be informative to directly quantify FOCM intermediates to evaluate possible alteration in the ratio of abundance of individual metabolites that may reflect disturbance of a particular step(s). For example, 10-formyl-THF provides one-carbon units for purine biosynthesis and a reduced abundance of this metabolite, relative to its precursor THF, could lead to diminished purine synthesis. Similarly, alteration in the relative abundance of 5-methyl THF may be informative about the contribution of methyl groups to the methylation cycle.

Folates are transported into cells in the monoglutamated form and multiple glutamate residues are then added by the action of folylpolyglutamate synthetase. Polyglutamation is required for cellular retention of folates and an optimal analytical approach should therefore distinguish these forms. Previously, we used liquid chromatography tandem mass spectrometry (LC–MS/MS) for quantification of *s*-adenosyl methionine (SAM) and *s*-adenosylhomocysteine (SAH) in order to identify perturbations in the methylation cycle [20, 21]. In the current study, we applied LC–MS/MS to analyse FOCM intermediates in bacteria and for comparison of human and mouse cell lines and embryonic tissue, which have not previously been subject to comprehensive analysis.

## Materials and methods

### Chemicals

Folate standards including dihydrofolate (DHF), tetrahydrofolate (THF), 5,10-methylene THF, 5-formyl-THF, 5,10-methenyl THF, 5-methyl THF, folic acid (PteGlu), PteGlu3, PteGlu4, PteGlu5, PteGlu7 and methotrexate were purchased from Schircks Laboratories (Switzerland). Remaining reagents were methanol, acetonitrile (Fisher Scientific, UK), ammonium acetate, ascorbic acid, DTT, citric acid (Sigma-Aldrich) and *N*,*N*-dimethylhexylamine (Fluka).

### Samples for analysis

*Bacteria* OP50 and HT115 *Escherichia coli* strains [22–24] were grown overnight in LB from a single colony at 37 °C. NGM plates [24] were seeded with 150 μL bacterial suspension and incubated for 96 h at 20 °C. Bacterial lawns were washed from the plates using M9, collected by centrifugation at 4 °C, 4,000 rpm for 20 min and the bacterial pellet stored at −80 °C prior to analysis. *Cell lines* EBV-transformed human lymphocytes were collected with ethical permission from normal Swedish individuals (Karolinska Institutet). Cells were cultured in RPMI 1640 media with 10 % FCS. For LC–MS/MS analysis, 2 × 10^7^ cells were harvested, washed in PBS and cell pellets stored at −80 °C prior to sample preparation. *Human tissue* was obtained as frozen samples (at −80 °C) from the human developmental biology resource (www.hdbr.org). *Mice* wild-type (CBA/Ca and C57BL/6) strain mice were mated and mouse embryos were collected at embryonic day (*E*) 12.5 [25]. Embryos were immediately frozen on dry ice and stored at −80 °C.

### Sample preparation

Buffer containing 20 mM ammonia acetate, 0.1 % ascorbic acid, 0.1 % citric acid and 100 mM DTT at pH 7 was added to cells, tissues or embryos. Where quantitation was performed, 3 μl of 10 μM methotrexate was added as internal standard. Sample suspensions were sonicated for 10 s using a hand-held sonicator at 40 % amplitude. A 10 μl aliquot of each of the homogenised samples was removed for DNA quantification using NanoDrop (thermo scientific). Protein was removed by precipitation by addition of 2 sample volumes of acetonitrile, mixing for 2 min and centrifugation for 15 min at 12,000×*g* and 4 °C. Supernatants were transferred to fresh tubes, lyophilised and stored at −80 °C prior to analysis.

### LC–MS/MS

Lyophilised samples were resuspended in 50 μl water (milli-Q) and centrifuged for 5 min at 12,000×*g* at 4 °C. Supernatants were transferred to glass sample vials for LC–MS/MS analysis. Metabolites were resolved by reversed-phase chromatography (Luna C18 column; 150 × 2.0 mm^2^; 5 μm bead size; Phenomenex, UK) using a 2795XE high performance liquid chromatography unit with solvent divert valve (Waters Corporation, UK). Solvents for HPLC were: Buffer A, 5 % methanol, 95 % Milli-Q water and 5 mM dimethylhexylamine at pH 8.0; Buffer B, 100 % methanol, 5 mM dimethylhexylamine. The column was equilibrated with 95 % Buffer A: 5 % Buffer B. The sample injection volume was 40 μl. The HPLC protocol consisted of 95 % Buffer A: 5 % Buffer B for 1 min, followed by a gradient of 5–60 % Buffer B over 9 min and then 100 % Buffer B for 6 min before re-equilibration for 4 min. The metabolites were eluted at a flow rate of 200 nl/min. The HPLC was coupled to a triple quadrupole tandem mass spectrometer (MicroMass Quattro, Waters Corporation, UK) operating in negative-ion mode using the following settings: capillary 3.54 kV, source temperature 150 °C, desolvation temperature 350 °C, cone gas flow rate 25 l/h and desolvation gas flow rate 950 l/h. Folates were measured by multiple reaction monitoring (MRM) with optimised cone voltage and collision energy for precursor and product ions (based on [26]).

### Data analysis and statistics

The peak areas of individual folates were extracted using MassLynx software (Waters). The total peak area of each folate (sum of mono- and polyglutamated forms) was expressed as a percentage of the total folate in each sample. Data were analysed using SigmaStat (Systat Software, Version 3.5). Pairwise comparisons were made by *t* test, and multiple comparisons by one way ANOVA with Holm-Sidak post hoc test.

## Results

In order to allow analysis of multiple folate species, we first determined optimal MRM conditions for detection of individual folate standards, based on reported MS parameters [26]. Thus, MRMs were determined for mono-glutamated forms of THF, 5-formyl-THF (CHO-THF), 5-methyl THF (5-CH_3_ THF), 5,10-methylene THF (5,10-CH_2_ THF), 5,10-methenyl THF (5,10-CH THF) and DHF, together with multiple glutamated forms of folic acid with 1, 3, 4, 5 and 7 glutamates (Table 1). These characteristic MRMs allowed individual analysis of each folate in a mixture of all the standards. Using the MRMs of polyglutamated folic acid and in comparison to previously reported values [26], we deduced MRMs for the polyglutamated forms of the various endogenous folates.

**Table 1:** MRM values for folate standards

Folate	MRM transition (*m*/*z*)
Monoglutamates	
5-Formyl THF	472.17 > 314.95
5-Methyl THF	458.19 > 328.89
5,10-Methylene THF	456.28 > 326.98
5,10-Methenyl THF	454.00 > 281.00
THF	444.20 > 314.85
DHF	441.97 > 175.99
Folic acid: mono and polyglutamates	
PteGlu (folic acid)	440.05 > 310.86
PteGlu3	698.37 > 421.99
PteGlu4	827.52 > 422.02
PteGlu5	956.67 > 422.03
PteGlu7	606.84 > 422.02

The MRM transitions (precursor > product ion) are indicated for each of the monoglutamated folate standards as well as polyglutamated forms of folic acid. Specific MRMs allow selective identification of each folate within a mixture of all the folates. Note the PteGlu7 precursor ion is doubly charged, hence the *m*/*z* value is lower than that for PteGlu3-5

**Fig. 2:**
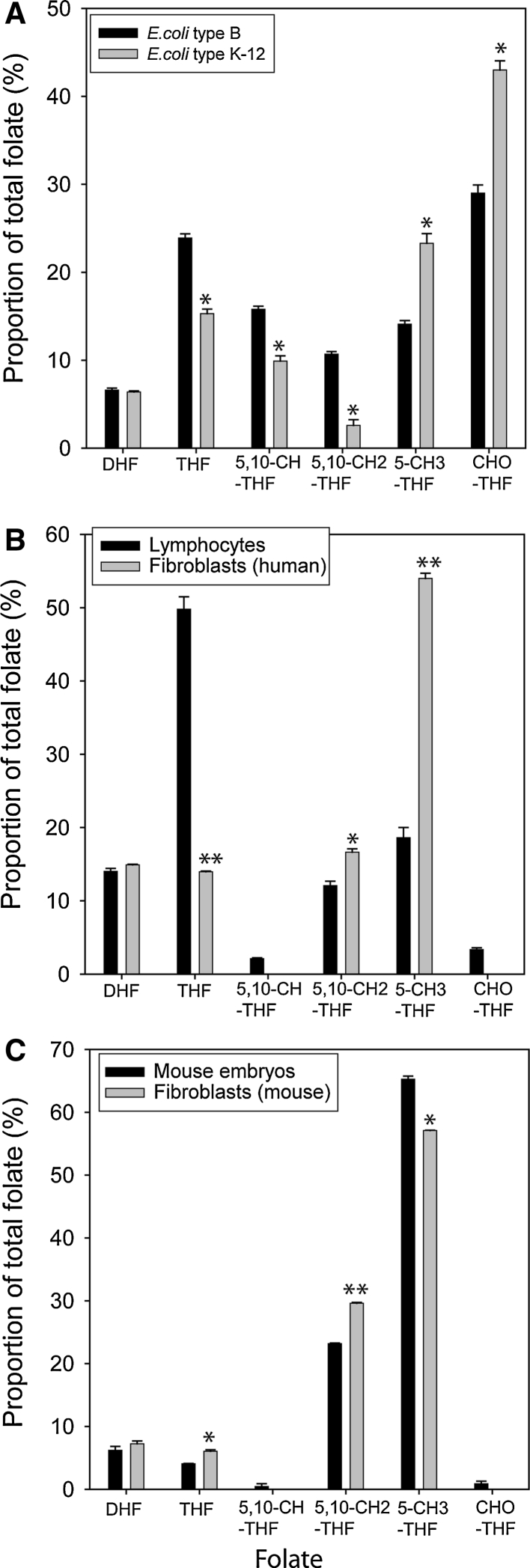
Folate profiles of human and mouse cell lines and embryos. The abundance of individual folates (sum of all glutamated forms) is expressed as a proportion of the total folate content in **a** bacteria, **b** human lymphoblastoid and primary fibroblast cell lines, and **c** primary mouse embryonic fibroblasts and mid-gestation (E12.5) embryos. **a** The relative abundance of all major folates differed between bacterial sub-types (*significantly different from corresponding folate in type B, *p* < 0.001). **b** Amongst human cell lines, there was a significant difference in the proportion of THF, 5,10-methylene THF and 5-methyl THF (**p < 0.001; *p < 0.02 difference in proportion compared with the corresponding folate in lymphocytes). The overall folate profile appeared similar between mouse embryos and fibroblasts but we observed significant differences in the proportion of THF, 5,10-methylene THF and 5-methyl THF (**p < 0.001; *p < 0.02 difference in proportion compared with the corresponding folate in embryos). Data are expressed as mean ± SEM; for bacteria, *n* = 10 replicates per analysis, for cell lines and embryos, *n* = 3–5 samples per group

Previous LC–MS/MS analysis of folate profiles in biological samples revealed differences in distribution between plants (spinach) and mammalian tissue (liver) [26]. In the current study, we analysed bacteria as well as mammalian cells and tissues, focussing on mouse and human embryonic material for which folate profile data have not previously been reported. Each of the major folate species was detectable in bacteria, the most abundant species being formyl-THF, THF and 5,10-methenyl THF (Fig. 2a). The bacterial strains analysed belong to different sub-groups, OP50 being derived from *E. coli* B and HT115 from *E. coli* K-12. With the exception of DHF, there was a significant difference between strains in the relative proportion of all the analysed folates (expressed as percentage of total folate). The OP50 strain had a higher proportion of THF, 5-methyl THF and 5,10-methylene THF than in HT115, and a lower proportion of 5,10-methenyl THF and formyl-THF (Fig. 2a).

**Fig. 3:**
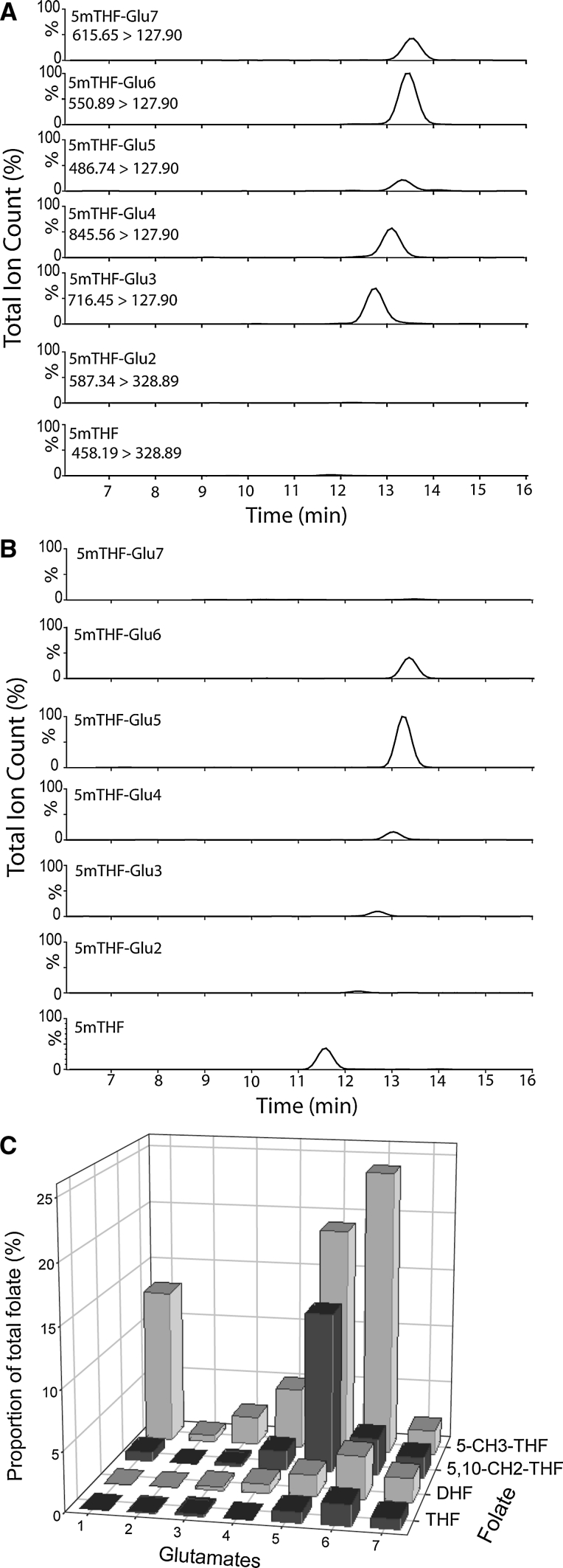
Analysis of polyglutamated folates in bacteria and mouse embryos. MS chromatograms are shown for 5-methyl THF (5mTHF) in mono-glutamated and polyglutamated form (with up to seven glutamates, Glu1-7) in **a** bacteria and **b** mouse embryo samples. Within each sample, the chromatograms are generated simultaneously with each glutamated form detected on the basis of the specific MRM. In addition, the *y*-axis (ion count) is linked to enable visualisation of relative abundance of each glutamated variant within a sample. The most abundant forms of 5-methyl THF are 3, 4 and 6 glutamated in bacterial samples and 5 or 6 glutamated in mouse embryo samples. **c** Similarly, comparison of the relative proportions of glutamated forms of 5-methyl THF, 5,10-methylene THF, DHF and THF in mouse embryos showed that in each case, the predominant forms were 5, 6 or 7 glutamated

In mammalian cell lines and tissue (Fig. 2b, c), the folate profile differed markedly from that observed in bacteria. The most notable difference in mammalian samples was the lower abundance of formyl-THF, which was virtually undetectable in some cell/tissue types. Analysis of primary fibroblasts derived from human foetal skin (Fig. 2b) and mouse embryonic fibroblasts (MEFs; Fig. 2c) revealed a similar overall pattern of the folate profile. However, the mean relative abundance of DHF, THF, 5,10-methylene THF and 5-methyl THF did significantly differ between mouse and human fibroblasts. The overall profile of folates in samples derived from mouse embryos at E12.5 was also similar to that of mouse and human fibroblastic cell lines. Despite the comparable trends, the differences in relative abundance of specific folates (THF, 5,10-methylene THF and 5-methyl THF) between mouse fibroblasts and embryos were statistically significant (Fig. 2c). For different cell or tissue types, the folate profile, in which the amount of each folate is expressed as a percentage of the total, enables comparison of the relative proportions of the folates which may reveal metabolic differences. Inclusion of an internal standard (either isotopically-labelled standard or folate analogue) also enables quantitation. For example, in mouse embryos and fibroblasts, we compared folate profiles generated using quantitative data (with normalisation to DNA content; Fig. S1b) with profiles for the same samples in which relative percentage of each folate was plotted (Fig. 3c and reproduced in Fig. S1a). In each case, the overall pattern of folate quantities was similar in fibroblasts and embryos, but abundance was lower in embryos than in fibroblasts (Fig. S1). In general, where overall folate quantity differs between tissues, the relative abundance profile may be most informative about metabolic flux [18].

In contrast to comparisons between fibroblasts and mouse embryos, the folate profiles of these cell and tissue types differed markedly from those observed in lymphoblastoid cell lines. The principal difference was the predominance of 5-methyl THF as the major folate species in fibroblasts and embryos, compared with THF in lymphoblastoid cells (Fig. 2b, c).

In addition to comparison of the total abundance of each major folate, we also analysed glutamation patterns. In bacterial samples, multiple glutamated forms of each folate including all types from Glu_2_ to Glu_7_ were present in varying proportions (Fig. 3a shows 5-methylTHF in OP50 strain as a representative example). The most abundant polyglutamated form in bacteria was somewhat variable between different folates, but in general corresponded to Glu_3_, Glu_4_, Glu_6_ and Glu_7_. The Glu_2_–Glu_7_ forms of 5-methyl THF were also detected in mouse embryos, the major forms being Glu_5_ and Glu_6_ (Fig. 3b)_._ However, in contrast to bacteria, the monoglutamate also made up a sizeable proportion (~20 %) of 5-methyl THF in embryos. Analysis of the glutamation state of several folates suggested that abundance of monoglutamate is principally a feature of 5-methyl THF, as the predominant forms of the other folates were the 5–7 glutamated forms (Fig. 3c).

**Fig. 4:**
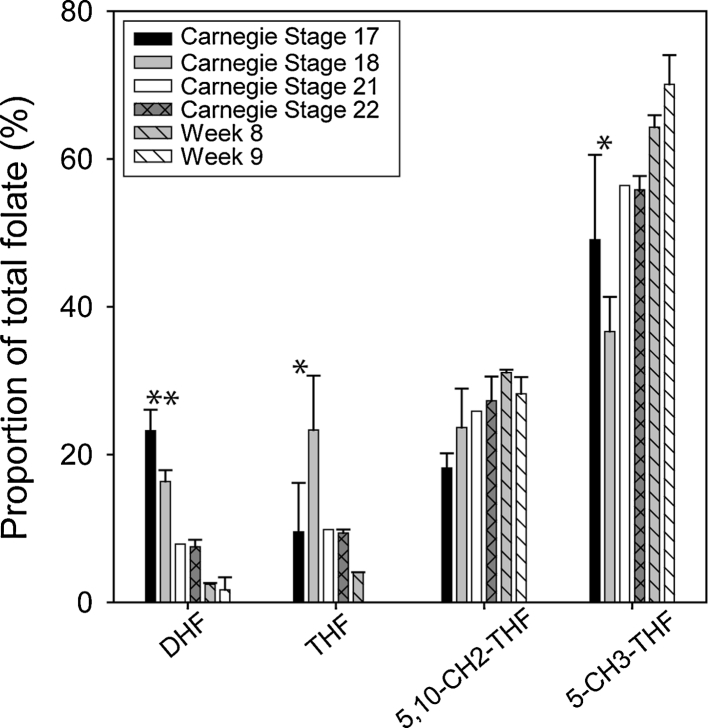
Comparison of folate profiles in human brain samples with increasing gestational age. Analysis of samples of embryonic brain at stages from CS 17 to week 9 revealed alterations in folate profile. DHF showed a decline in relative abundance (**indicates significant difference of CS 17–18 to CS 21–22 and week 8–9; *p* < 0.001) as did THF, whilst 5-methyl THF increased in relative abundance through this period of development (*indicates significant difference between CS 17–18 and week 8–9; *p* < 0.001). Data are expressed as mean ± SEM; *n* = 2 CS 17, 3 CS 18, 1 CS 21, 2 CS 22, 2 week 8 and 2 week 9 samples, each from a separate individual

To date little data is available on folate metabolism in human tissue during development. Therefore, we also analysed tissue samples derived from human embryonic and early foetal brain, in a series of developmental stages from Carnegie stage (CS) 17 (~6 weeks) to 9 weeks of gestation (Fig. 4). The largest change in folate profile with increasing gestational age was a decline in the abundance of DHF, which made up a significantly larger proportion of total folate in CS 17–18 brain samples (stages combined for comparison purposes) compared with CS21-22 and Week 8–9 (*p* < 0.001 ANOVA). The proportion of THF also declined with developmental stage, whereas 5-methyl THF showed an overall increase in contribution to the overall folate pool (Fig. 4). In both cases, there was a significant difference in proportion between CS 17–18 and Week 8–9 samples (*p* < 0.001). Amongst the stages analysed, the folate profile at CS 21–22 was most similar to that observed amongst human and mouse fibroblasts or mouse embryos at E12.5 (compare Figs. 2, 4).

**Fig. 5:**
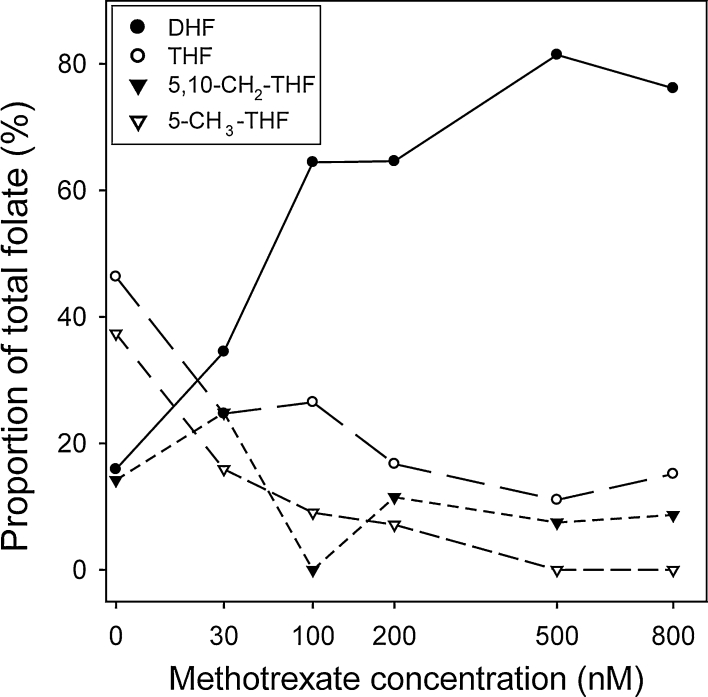
Methotrexate causes altered folate profile in lymphoblastoid cell lines. Cells were cultured in a series of concentrations of methotrexate (scale of the *x*-axis is based on log value of the indicated methotrexate dose). LC–MS/MS showed a dose-dependent increase in the relative abundance of DHF, correlating with decline in abundance of THF and 5-methyl THF

Having compared folate profiles amongst different cell types and species, we investigated systems in which FOCM is compromised. We tested the ability of the LC–MS/MS methodology to detect alterations of folate profile, by cultured human lymphoblastoid cell lines in the presence of a varying concentration of methotrexate, an inhibitor of dihydrofolate reductase (DHFR; Fig. 1). We observed a dose-dependent elevation of DHF, with a corresponding decrease in abundance of the other major folates, particularly 5-methyl THF (Fig. 5). These findings correspond with the response to DHFR inhibition predicted by computational modelling of FOCM [27, 28].

## Discussion

Folate one-carbon metabolism plays a fundamental role in key cellular functions including nucleotide biosynthesis and methylation and is implicated in a number of diseases. Comprehensive profiling of folates within a cell or tissue type will provide an opportunity to investigate disturbance of metabolism and potentially to focus attention on particular step(s) that may be abnormal in a disease state or following treatment with pharmacological agents. Moreover, folate profiling will provide opportunities to investigate the metabolic effects of targeted mutation of FOCM enzymes in model systems. This may include loss-of-function alleles, a number of which have already been generated in mice, as well as specific alleles designed to mimic putative disease-causing mutations in humans.

The sensitivity of LC–MS/MS profiling to detect gross disruption of FOCM was tested by exposure of lymphoblastoid cells to methotrexate, a DHFR inhibitor. As predicted, the level of DHF was markedly increased in treated cells, a finding which parallels the elevation in DHF levels observed in lymphoblasts derived from patients with inherited DHFR deficiency [29].

LC–MS/MS methodology has previously been used for simultaneous profiling of folate metabolites in samples from different plant species [26]. In the current study, we used an LC–MS/MS approach to perform comprehensive folate profiling in bacterial sub-types as well as mouse and human cell lines and embryonic tissue. Each of the analysed folates was detectable in each of the different types of samples but we observed notable variation in the relative abundance of the different folate metabolites. Perhaps unsurprisingly, the overall folate profile substantially differed between bacterial and mammalian samples, in particular with a greater proportion of formyl-THF in bacteria. These differences may reflect, in part, the variation in FOCM enzymes in bacteria and mammalian cells. In particular, in eukaryotes, the trifunctional enzyme MTHFD1 (methylene tetrahydrofolate dehydrogenase/methylene tetrahydrofolate cyclohydrolase/formyltetrahydrofolate synthetase) catalyses the interconversion of 5,10-methylene THF, 5,10-methenyl THF and 10-formyl-THF [1, 2]. However, in bacteria, these reactions are catalysed by two or three separate enzymes with conversion of THF to 10-formyl-THF by a monofunctional synthetase enzyme. In addition to possible variation in regulation of FOCM, differences in folate profile likely reflect the fact that, unlike mammalian cells, bacteria can synthesise folates de novo.

The overall folate profile patterns were similar in human and mouse fibroblast cell lines and mouse embryos, despite differences in the relative abundance of specific folate metabolites. In contrast, lymphoblastoid cell lines exhibited a rather different profile, with a significantly higher THF and lower 5-methyl THF than in fibroblasts. The metabolic basis of this variation in profiles has not been defined but computational modelling indicates that reduced activity of MTHFR or elevated activity of methionine synthase in lymphoblasts compared with fibroblasts could result in the observed difference in profile. The functional significance of differing profiles could be hypothesised to reflect the metabolic requirements of different cell types. For example, diminished MTHFR activity could indicate channelling of one-carbons towards nucleotide biosynthesis at the expense of methylation in lymphoblasts.

We also observed developmental shifts in folate profile in human embryonic brain, with an overall decline in DHF and THF and a corresponding increase in 5-methyl THF during the period from around 6 to 9 weeks of gestation. This change correlates with the onset of neuronal migration in the primate embryonic brain [30] and could potentially be effected through increased activity of MTHFR (or reduced activity of methionine synthase) with development. Increased availability of 5-methyl THF could potentially support methylation reactions at later stages as the overall behaviour of the cellular population shifts from proliferation towards differentiation.

In recent years, it has become increasingly clear that FOCM is compartmentalised at the sub-cellular level and that this is likely to be functionally important in regulation of flux through different pathways [2, 31]. For example, enzymes required for de novo thymidylate biosynthesis (SHMT1, TYMS and DHFR) localise to the nucleus during S and G_2/_M phase of the cell cycle [31]. Folates must, therefore, be present in the nucleus to supply one-carbon units for dTMP synthesis. Mitochondrial folate metabolism plays a crucial role in provision of one-carbon units as formate, to the cytoplasm (and nucleus). Thus, the majority of one-carbons used for nucleotide biosynthesis and methylation are derived from serine, glycine, dimethylglycine or sarcosine in mitochondria [2]. Mitochondrial-specific FOCM-related enzymes include MTHFD1L and MTHFD2L and the glycine cleavage system (GCS). Mutations in *GLDC* or *AMT*, encoding components of the GCS, cause non-ketotic hyperglycinemia and predispose to NTDs [32, 33]. The identification of diseases associated with FOCM in specific sub-cellular compartments suggests that fractionation prior to LC–MS/MS analysis will be important in evaluating the metabolic consequences of putative causative mutations. Comprehensive metabolite profiling, in combination with predictive modelling, may then provide a useful tool to investigate downstream functional effects on FOCM and indicate possible routes to therapeutic intervention.

## Electronic supplementary material

Below is the link to the electronic supplementary material.

Supplementary material 1 (PDF 178 kb)
