# Supplementary material for: Folate metabolite profiling of different cell types and embryos suggests variation in folate one-carbon metabolism, including developmental changes in human embryonic brain
Source: Mol Cell Biochem. 2013 Mar 13;378(1):229–36. doi: 10.1007/s11010-013-1613-y (PMC3634978; doi:10.1007/s11010-013-1613-y)
Supplement: Supplementary file 1 — Supplementary material 1 (PDF 178 kb) [file 11010_2013_1613_MOESM1_ESM.pdf]

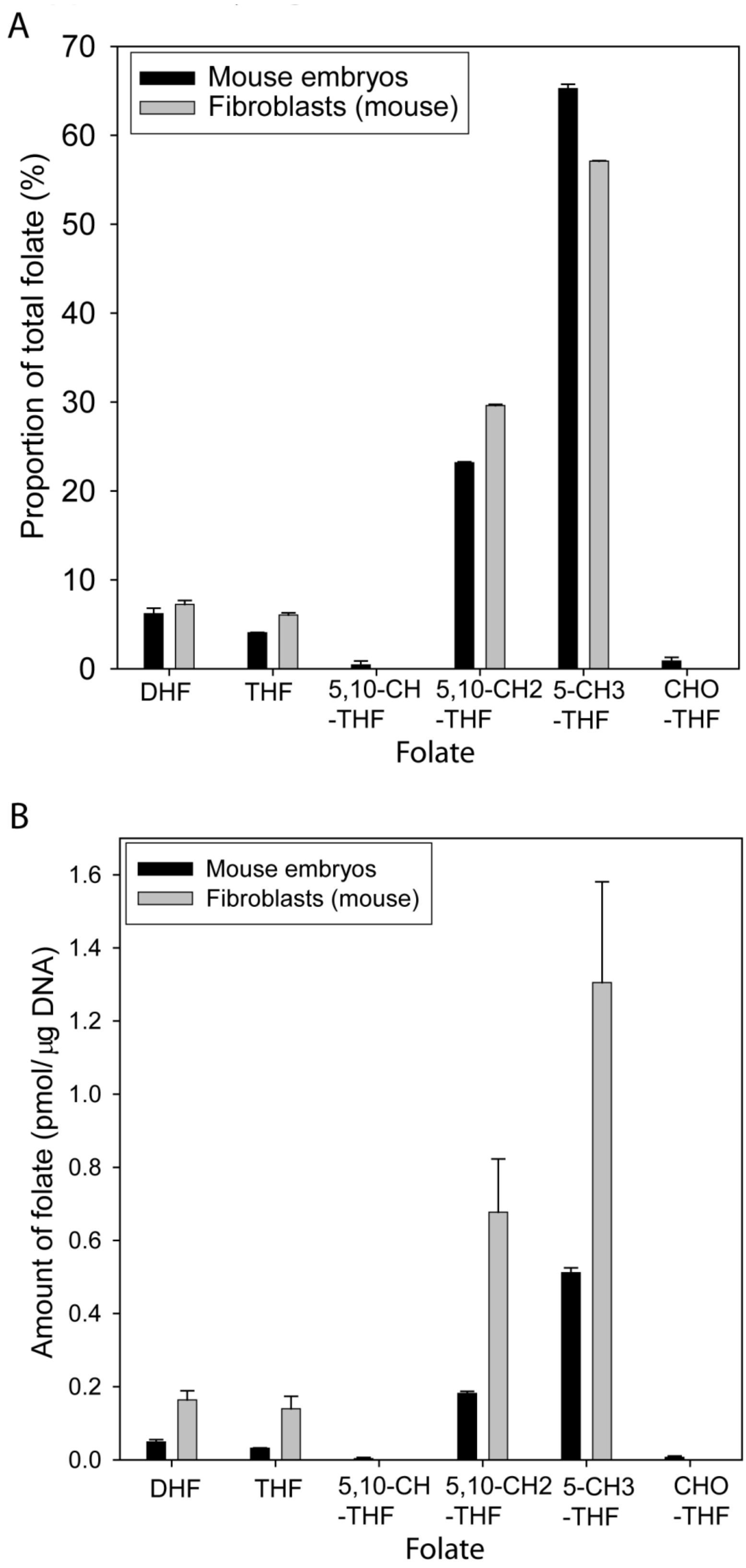

Supplementary Material Figure S1. Folate profiles of E12.5 mouse embryos and primary mouse embryonic fibroblasts. The abundance of individual folates (sum of all glutamated forms) is expressed as (A) proportion of the total folate content (reproduced from Fig 2C) or (B) amount of folate, normalised to DNA content of the sample (using methotrexate as internal standard). Both profiles show a similar relative abundance of folate types within a sample (more readily apparent in A), while absolute abundance of folates is lower in embryo samples than in fibroblasts (B).
